# Resilience status of dental students and derived training needs and interventions to promote resilience

**DOI:** 10.3205/zma001649

**Published:** 2023-11-15

**Authors:** Mia T. Schwitters, Jan Kiesewetter

**Affiliations:** 1 Technical University Dresden, Faculty of Medicine Carl Gustav Carus, Dresden, Germany; 2 LMU Hospital, LMU Munich, Institute of Medical Education, Munich, Germany

**Keywords:** dental school, resilience, burnout, stress, medical education

## Abstract

**Background:**

The concept of resilience is defined differently in the literature, with the definition depending on the criteria under consideration. Currently, the most commonly used definition is: resilience as “psychological resistance to biological, psychological, and psychosocial developmental risks”. In order to systematically enhance resilience, it is necessary to first determine specific training needs. This study examines the resilience status of dental students in Germany from different academic years and derives interventions for resilience enhancement, as the field of dentistry is considered one of the “most stressful professions”.

**Methods:**

To determine the resilience status, a questionnaire was developed, consisting of the 10-Item Connor-Davidson Resilience Scale (10-Item CD-RISC), the Maslach Burnout Inventory Scale (MBI), the Negative Self-Image Scale (NSBS), and five self-formulated closed-ended questions. A total of 320 questionnaires were distributed, with 184 responses (43.7% female) received, including partially completed forms.

**Results:**

The resilience status shows an average moderate level of resilience (*M*=28.43; *SD*=5.57). The subcomponents of* emotional exhaustion* (*M*=23.66; *SD*=8.32) and *reduced personal performance* (*M*=33.69; *SD*=8.47) indicate an increased risk of burnout, but not *depersonalization* (*M*=5.04; *SD*=5.50). Overall, the participants have a positive self-image (*M*=1.72; *SD*=0.69).

**Conclusion:**

The study reveals that dental students have a moderate level of resilience. Dental students are not inherently prone to burnout, but they show reduced levels of emotional exhaustion and personal performance, suggesting a need for interventions in these areas. Possible interventions tailored to these training needs are discussed in the article. Further research is needed to determine the effectiveness of these interventions.

## Zusammenfassung

**Hintergrund:**

Der Begriff der Resilienz wird in der Literatur unterschiedlich definiert. Dabei hängt die Definition von den betrachteten Kriterien ab. Heutzutage wird von folgender Definition am häufigsten Gebrauch gemacht: Resilienz als „die psychische Widerstandsfähigkeit gegenüber biologischen, psychologischen und psychosozialen Entwicklungsrisiken“. Damit die Resilienz zielorientiert gesteigert werden kann ist vorher die Frage zu klären, welche spezifischen Trainingsdesiderate bestehen. Die Studie untersucht den Resilienzstatus von Zahnmedizinstudierenden deutschlandweit aus unterschiedlichen Jahrgangsstufen und leitet daraus Interventionen zur Förderung von Resilienz ab, denn das Fachgebiet der Zahnmedizin gehört zu den „stressreichsten Professionen“.

**Methoden:**

Für die Bestimmung des Resilienzstatus wurde ein Fragebogen erstellt, der sich aus der 10-Item Connor-Davidson Resilience Scale (10-Item CD-RISC), der Maslach Burnout Inventory Skala (MBI), der Negatives Selbstbild-Skala (NSBS) und fünf selbst gewählten geschlossenen Fragen ergibt. Es wurden insgesamt 320 Fragebögen mit einem Rücklauf von 184 (43,7% weiblich) verteilt. Dabei wurden auch unvollständig ausgefüllte Bögen berücksichtigt.

**Ergebnisse:**

Der Resilienzstatus zeigt eine durchschnittlich moderate Resilienz auf (*M*=28,43; *SD*=5,57). Die Subkomponenten *emotionale Erschöpfung* (*M*=23,66; *SD*=8,32) und *reduzierte persönliche Leistungsfähigkeit* (*M*=33,69; *SD*=8,47) weisen auf ein erhöhtes Burnout-Risiko hin, jedoch nicht die *Depersonalisation* (*M*=5,04; *SD*=5,50). Insgesamt verfügen die Teilnehmer*innen über ein gutes Selbstbild (*M*=1,72; *SD*=0,69).

**Schlussfolgerung:**

Die Studie zeigt für Zahnmedizinstudierende eine Resilienz im mittleren Bereich. Dabei sind Zahnmedizinstudierende nicht per se Burnout gefährdet, jedoch in den Subkomponenten emotionale Erschöpfung und reduzierte persönliche Leistungsfähigkeit so reduziert, dass hier Interventionen erfolgen sollten. Mögliche auf diese Trainingsdesiderate zugeschnittene Interventionen werden im Artikel diskutiert. Weitere Forschung ist notwendig, um zu überprüfen, ob die entsprechenden Interventionen zielorientiert wirken.

## 1. Background

Due to contemporary societal changes, there often is limited time for individuals to recuperate their own resources [11]. Students, particularly those in medical programs, face high levels of psychological stress [4]. Numerous studies have examined the burnout risk in medical students [6]. The average prevalence of burnout in these studies is reported at 44%. Recent research has also focused on resilience, defined as “psychological resistance to biological, psychological, and psychosocial developmental risks” [22]. First-year medical students, in particular, have reported encountering problems when dealing with their own emotions in challenging situations [10]. Hence, it is crucial to further explore resilience and develop methods to manage challenging situations in a way that prevents medical students from experiencing burnout [4], [11]. Nowadays, resilience is viewed as a “dynamic process of adaptation and development” [22], making targeted training and intervention research essential.

Dentistry programs are considered among the “most stressful professions”, necessitating examinations of burnout risk and resilience in dental education [21]. However, compared to medical students, there are significantly fewer studies on dental students in the literature. Given the scope of dental education, one might assume a similar level of stress as in medical education. Nevertheless, dental education incorporates more practical aspects, often incorporating direct patient interactions, and may require different interventions. Approximately 20% of dental students exhibit moderate levels of depression [8]. Due to differences in the structure of their curricula, dental students face increased performance pressure and potential stressors [12]. Half of the students lack stress management strategies, leading to negative lifestyle consequences such as increased caffeine consumption, lack of physical activity, and a significant proportion of smokers [8], [19]. Opportunities for resilience, however, are rarely addressed [13]. To systematically enhance resilience, it is essential to first determine specific training needs. This study examines the resilience status of dental students nationwide in Germany and deducts interventions to promote resilience. To identify specific training needs, essential components including burnout, resilience, and participants’ self-image will be examined.

## 2. Materials and methods

Data was collected from dental students across all academic years in Germany. To maximize the sample size, in the first data collection phase on November 9, 2019, students were selected during the general assembly of the Federal Student Council for Dental Students in Halle (recruitment 1). Students from all faculties were represented at the conference by the respective members of their faculty’s student council. Additional surveys of dental students were conducted at the Technical University of Dresden on November 18, 2019 (recruitment 2). In total, 270 (recruitment 1) + 50 (recruitment 2) questionnaires were distributed.

The questionnaire for the first data collection phase was based on three validated scales for measuring resilience. The first instrument used was the German version of the 10-Item Connor-Davidson Resilience Scale (10-Item CD-RISC) [18]. It has been validated as an effective tool for measuring resilience [2] and exhibits the “best psychometric properties” [18]. Participants were asked to respond to ten statements on a scale from 0 (not true at all) to 4 (almost always true), with all statements formulated positively. This was followed by the Negative Self-Image Scale (NSBS) [17], which allowed for the quantitative assessment of participants’ negative self-perception [16], and the German version of the Maslach Burnout Inventory Scale (MBI) [5]. The NSBS consists of 27 negative statements to be rated on a scale from 1 (not worried at all) to 5 (extremely worried). The MBI comprises 22 positive or negative statements about possible emotions and thoughts, with participants indicating frequency (1=several times a year to 6=daily) and intensity (1=very weak/barely noticeable to 7=significant, very strong).

In addition to these scales, five self-formulated questions in a closed-ended format were included, as well as a demographic data survey (age, gender, academic semester).

For clarity and conciseness, the introductory descriptions of all three scales were shortened in the questionnaire, focusing only on the methodology. In total, the final questionnaire consisted of 64 items divided into four sections. All participants were informed of the survey’s purpose and the anonymity of their data. Participants consented to data processing. A brief introduction to the study was provided before distributing the questionnaire to the participants. Ethical approvals were obtained from the ethics committees of the medical faculty at LMU Munich and the medical faculty at TU Dresden.

Data processing was performed using Microsoft Excel 365 and SPSS 25. It was ensured that the NSBS [17] and the MBI [5] consisted of subcomponents, with sums and means calculated for each.

Since the MBI contains both positively and negatively formulated items, positively formulated items were recoded to enable consistent interpretation of values. Descriptive statistics, including relative and absolute frequencies, median, mean, standard deviation, variance, minimum, and maximum, were calculated. Multivariate analysis of variance was conducted to examine the relationships between the scale results and the recruitment locations and gender.

## 3. Results

The response rate was 60.37% (*N*=163) for recruitment 1 and 42% (*N*=21) for recruitment 2. After data verification, no significant differences between the two were identified, possibly due to the varying number of fully completed questionnaires. Therefore, the results were collectively analyzed (see table 1 (Tab. 1)).

The age of participating students ranged from 18 to 33 years, with an average age of 23.4 years (*SD*=2.93). In terms of gender, participants were 56.3% male and 43.7% female, with an average of the sixth academic semester (*M*=6.3; *SD*=2.22), representing all eleven academic semesters. The fifth and seventh semesters were the most common (*N_5_*= 43, *N_7_*=49). Table 2 (Tab. 2) provides an overview of the distribution of academic semesters.

The 10-Item CD-RISC has a maximum possible sum score of 40 points. Based on this classification, the participants in the study exhibited moderate resilience (*M*=28.43; *SD*=5.57). The Maslach Burnout Inventory Scale was evaluated in its subcomponents. The participating students displayed a slightly increased risk of burnout in terms of the subcomponents emotional exhaustion (*M*=23.66; *SD*=8.32) and reduced personal performance (*M*=33.69; *SD*=8.47), but not depersonalization (*M*=5.04; *SD*=5.50) (see table 3 (Tab. 3)).

Table 4 (Tab. 4) provides the sum mean values of the NSBS subcomponents. These sum mean values do not provide specific insights into participants’ responses to individual statements. Considering the varying number of items (see table 4 (Tab. 4)), the following means for individual items are obtained.

It can be inferred that participants have a positive self-image in all three areas covered by the NSBS, as the scale uses a range from 1 (not worried at all) to 5 (extremely worried).

Closed-ended questions are not detailed in the article.

## 4. Discussion

When comparing the results with those of medical students at German universities [10], dental students exhibit lower resilience on the 10-Item CD-RISC, with medical students having higher resilience (*M*=37.1; *SD*=0.63). According to the categorization by Notario-Pacheco et al. [15], resilience can be considered moderate but not high. In conjunction with the results from the Maslach Burnout Inventory Scale and the Negative Self-Image Scale, it appears that students generally possess resilience but require targeted training. The emphasis here lies in emotional exhaustion and reduced personal performance. This aligns with previous studies that identified a notable deficit in emotional exhaustion among dental students at the University of Dresden [8]. The surveyed sample should be critically assessed, as motivated students who may have higher resilience could be more prevalent at the Federal Student Council conference. This could be investigated further in a subsequent study.

The resilience training by Kiesewetter and Dimke [9] resulted in a significant improvement in the MBI Scale for medical students. Significant improvements were observed in the subcomponents emotional exhaustion (EE) and reduced personal performance (PA) [10]. These components are crucial for personal development because stressors do not end with the completion of education but persist in their future careers with challenging working conditions [8].

Dental professionals report higher job satisfaction, but many experience exhaustion after a day of work with insufficient recovery on weekends. High concentration and strong problem-solving skills are required during work to meet individual patient needs despite a high workload [14]. This suggests that such training may also have a positive effect on dental students, especially considering that the average resilience of dental students (M=28.4) is lower than that of medical students (*M*=37.1) [10]. Consequently, this study proposes the application of Resilience Training by Kiesewetter and Dimke [9] and its implementation within a doctoral project at the Technical University of Dresden. All planned training modules will be introduced to the students, divided into strategies for time and energy management (module 1), mindfulness (module 2), performance emotions (module 3), coping with setbacks and strong emotions (module 4), and work-life and life-work balance (module 5) [9]. Participation will initially be voluntary, with students from different academic semesters applying. The individual sessions will be scheduled based on the participants’ varying class schedules.

To positively influence the components of emotional exhaustion and reduced personal performance (PA), the modules focusing on time/energy management, performance emotions, and coping with setbacks (modules 1, 3, and 4) are particularly suitable. The first module addresses the relationship between time and resource management. Participants will learn that conscious management of their energy can lead to effective time management. The focus will be on the areas of body, emotions, mind, and spirit, along with corresponding mindful strategies [9]. In modules 3 and 4, participants will learn to manage performance emotions and strong emotions that can arise during their studies and future careers. They will explore their own (performance) emotions and learn to recognize them consciously. This will help them develop strategies to approach tasks with more motivation and to better handle strong emotions and setbacks. Initially, it is important to accept that there are both positive and challenging emotions. These emotions are not unusual or negative but part of the emotional spectrum that participants will learn to handle and practice [9].

To evaluate whether the individual training modules have differentiated effects on the measured components of emotional exhaustion and reduced personal performance, the questionnaire presented in this study is planned for use.

## 5. Conclusion

Dental students also require the focus of resilience studies to actively maintain their health, minimize burnout risks, and expand dental education to include the aspect of mental health maintenance. This study strongly emphasizes the need for training.

## Competing interests

The authors declare that they have no competing interests. 

## Figures and Tables

**Table 1:**
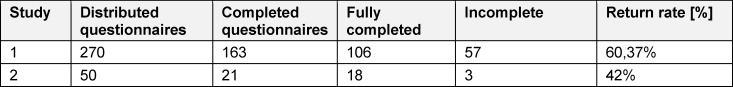
Return rates of questionnaires for studies 1 and 2

**Table 2:**
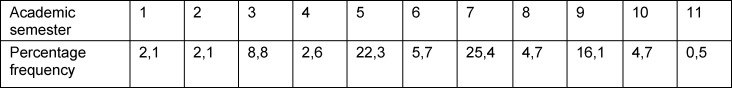
Distribution of academic semesters

**Table 3:**
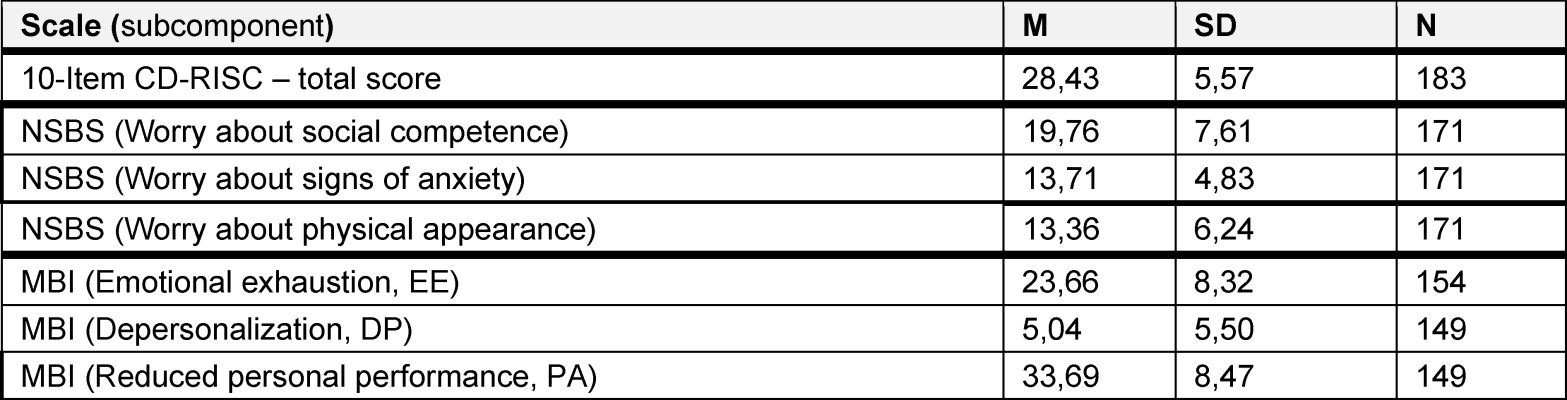
Sum mean values of the used scales

**Table 4:**
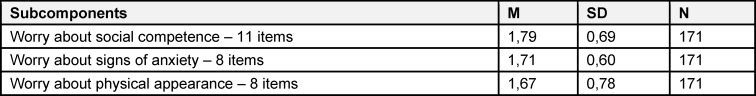
Mean values of NSBS subcomponents

## References

[1] Bengel J, Meindes-Lücking F, Rottmann N (2009). Schutzfaktoren bei Kindern und Jugendlichen. Stand der Forschung zu psychosozialen Schutzfaktoren für Gesundheit. Forschung und Praxis der Gesundheitsförderung. Band 35.

[2] Campbell-Sills L, Stein MB (2007). Psychometric analysis and refinement of the connor–davidson resilience scale (CD-RISC): Validation of a 10-item measure of resilience. J Trauma Stress.

[3] Claußen J (2022). Diamanten entstehen unter Druck... Freie Zahnarzt.

[4] Daniel-González L, García Cadena CH, Valle A, Caycho-Rodriguez T (2020). Validation study of the 10-item Connor-Davidson Resilience Scale among mexican medical and psychology students. Rev Psicología Ciencias Comportamiento.

[5] Enzmann D, Kleiber D (2004). Helfer-Leiden: Streß und Burnout in psychosozialen Berufen.

[6] Frajerman A, Morvan Y, Krebs MO, Gorwood P, Chaumette B (2019). Burnout in medical students before residency: a systematic review and meta-analysis. Eur Psychiatry.

[7] Huber-Metz B (2015). Kernkompetenz Resilienz. Sozialwirtschaft.

[8] Jurkat H, Höfer S, Richter L, Cramer M, Vetter A (2011). Lebensqualität, Stressbewältigung und Gesundheitsförderung bei Studierenden der Human- und Zahnmedizin - Eine Vergleichsuntersuchung. Dtsch Med Wochenschr.

[9] Kiesewetter J, Dimke B (2018). Resilienztraining für Studierende der Medizin, Ärzte & Gesundheitsfachpersonal.

[10] Kiesewetter J, Huber J (2021). A primer of an in-depth resilience status for German medical graduates: results of a cross-sectional survey on the status quo of resilience among graduates of human medicine in Bavaria, Germany. BMC Med Educ.

[11] Lieb K, Kunzler AM (2018). Resilienz. Nervenarzt.

[12] Liedl M (2007). Stressprofilanalyse bei Zahnmedizinstudenten im Vergleich mit Humanmedizinstudenten an der Universität Heidelberg unter Verwednung des Trierer Inventar zum chronischen Stress.

[13] Mache S, Vitzhum K, Groneberg D (2015). Prevention of study-related stress symptoms: health-promoting behavior among dental students. Wien Med Wochenschr.

[14] Micheelis W (2010). Zahnärzte im Arbeitsstress. Zahn Mitteilung.

[15] Notario-Pacheco B, Solera-Martínez M, Serrano-Parra MD, Bartolomé-Gutiérrez R, García-Campayo J, Martínez-Vizcaíno V (2011). Reliability and validity of the Spanish version of the 10-item Connor-Davidson Resilience Scale (10-Item CD-RISC) in young adults. Health Qual Life Outcomes.

[16] Richter AK (2019). EMDR bei Sozialen Angststörungen.

[17] Richter AK (2018). NSBS: Negatives Selbstbild-Skala. 1. Arbeitsversion. Deutsche Bearbeitung von Moscovicg DA, Huyder V (2012). Negative Self-Portrayal Scale (NSPS).

[18] Sarubin N, Gutt D, Giegling I, Bühner M, Hilbert S, Krähenmann O, Wolf M, Jobst A, Sabaß L, Rujescu D, Falkai P, Padberg F (2015). Erste Analyse der psychometrischen Eigenschaften und Struktur der deutschprachigen 10- und 25-Item Version der Connor-Davidson Resilience Scale (CD-RISC). Z Gesundheitspsychol.

[19] Stößel U (2005). Medizinstudierende - Eine Zielgruppe für Gesundheitsförderung an der Hochschule?. Gesundheitswesen.

[20] Wellensiek SK (2011). Handbuch Resilienz-Training.

[21] Wissel C, Wannemüller A, Jöhren H (2012). Burnout bei Zahnärzten - Ergebnisse einer bundesweiten Onlinebefragung in Deutschland. Dtsch Zahn Z.

[22] Wustmann C (2016). Resilienz. Widerstandsfähigkeit von Kindern in Tageseinrichtungen fördern.

